# Sustainable Optimization of Biotechnology for Cu Recovery from Printed Circuit Boards

**DOI:** 10.1021/acsomega.5c03870

**Published:** 2025-09-03

**Authors:** Alessandro Becci, José Miguel Rodríguez-Maroto, Juan Manuel Paz-Garcia, Francesca Beolchini, Alessia Amato

**Affiliations:** † Department of Life and Environmental Sciences, 9294 Università Politecnica delle Marche, Via Brecce Bianche, Ancona 60131, Italy; ‡ Department of Chemical Engineering, 16752 University of Malaga, Malaga 29071, Spain

## Abstract

The increase of waste from electrical and electronic equipment (WEEE), rich in valuable elements, has pushed research toward the development of sustainability treatments for its exploitation. The high Cu concentration within printed circuit boards (PCBs) (around 20–25% w/w) makes them a promising secondary resource. The aim of this work is the optimization of the patented bioleaching process driven by the minimization of the environmental load in the global warming category. The first environmental assessment was carried out considering bioleaching in a stirred tank reactor, also including metal recovery operations, using the best conditions identified in previous works. A mathematical model for bioleaching prediction was integrated inside the LCA methodology and used in Monte Carlo simulations: this assessment highlighted the energy demand as the main criticality. Consequently, a fixed bed column leaching was investigated both experimentally and theoretically through a predictive model, starting with the chemical leaching with Fe^3+^ and recirculation, as preparatory to our Copper BIOTECH patented technology. The excellent agreement among the predictive model and the experimental data provided a powerful tool for optimization purposes. The mathematical model was applied to patented technology, and simulations allowed us to identify the best operative conditions in bioleaching. The innovative design allows for a decrease of around 70% of energy demand and around 55% of the impact in the global warming category. Our study is fundamental to boost the application of sustainable bioleaching technologies in the world. Moreover, our methodological approach represents a guideline to meet sustainability goals within circular economy approaches for strategic metals.

## Graphical abstract

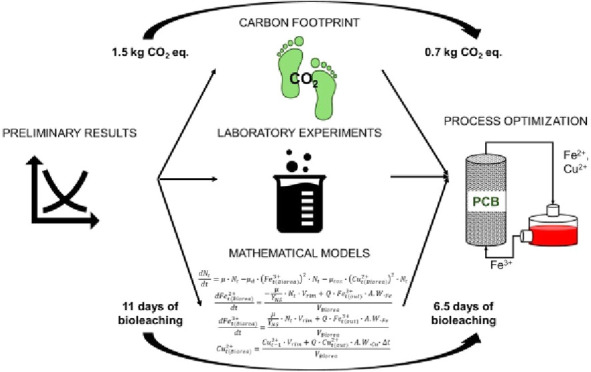

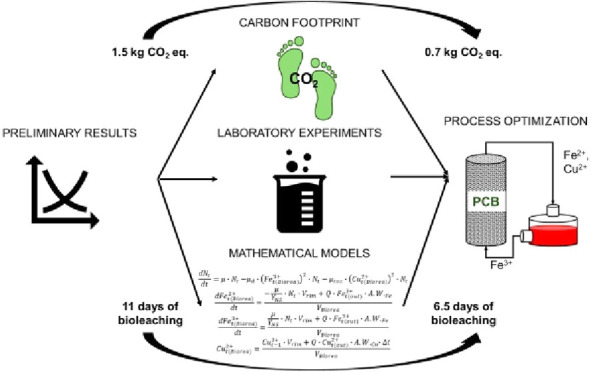

## 1 Introduction

In the last decades, municipal waste generation has increased rapidly, with a particular contribution of waste from electrical and electronic equipment (WEEE). WEEE constitutes 8% of the total municipal waste, with a world generation of around 62 million tons in 2022.^1–3^ The reason for the fast growth of WEEE is the lifespan decrease of electric and electronic equipment, mainly due to technological advancement and marketing strategies.^4–6^ The average generated WEEE per inhabitant in the world is 7.8 kg/year, and the highest value per inhabitant is recorded in Europe (17.6 kg/year).^3,7–9^

Printed circuit boards (PCBs) are the brain part of EEE, and they constitute 36% of the WEEE amount.^10–12^ PCBs are constituted by three main layers: the structure (composed of fiberglass and Cu), and upper and lower compound units (composed of fiberglass, solder joints, conductive tracks, contacts, and solder mask).^1^ Typically, the average content of metals, ceramics, and plastic is estimated of about 40, 30, and 30% (w/w), respectively.^8,13–16^ As concerns metal content, PCBs are composed of 20% Cu, 5% Al, 1% Ni, 1.5% Pb, 2% Zn, and 3% Sn (w/w) with approximately 250 Au, 1000 Ag, and 110 Pd (ppm).^1,7,10,17,18^

The PCB “mineralogy” is completely different from that of mineral ores for metal refining. Natural ores are composed of oxidized/sulfured minerals, while PCBs contain either pure metals or metal alloys in their structure.^1^ The Cu concentration in PCBs is 20–40 times higher than the concentration reported in minerals.^19–22^ Therefore, the potential value of metal recovery from PCBs is estimated between 1 and 25 €/kg. Cu is the most relevant valuable metal which can be recovered from PCBs, after Au and Pd.^1,5,23,24^

Around 2 Mt of Cu is used in Europe every year. Around 48% is imported by countries outside Europe, such as Chile, Peru, and China. Cu is mainly used as an electrical conductor, being the best conductor after silver, and it is an essential component for key technologies such as hybrid and electric cars and wind and solar power. For these reasons, Cu is included in the list of the strategic raw materials for the European Union since 2023.^25^

In this context (the huge amount of waste generated and the highest Cu demand for new device production), PCBs are considered an important source of Cu, and their recycling is very strategic. Considering the different metal speciation of Cu in PCBs with respect to mineral ores, the technologies for metal extraction from PCBs are different than those for mineral leaching.^16,26^ After collection and presorting, PCBs are pretreated (e.g., dismantling and size reduction), preprocessed (e.g., separation and metal extraction), and finally end-processed (e.g., purification and refining).^1,14^

Currently, there are some industries that treat and recover metals from PCBs, including Umicore (Belgium), Noranda (Canada), Rönnskär (Sweden), Dowa (Japan), and Aurubis (Germany).^1,27,28^ The main approaches used in these facilities for metal extraction are the pyrometallurgical and hydrometallurgical techniques. Pyrometallurgical processes need a temperature above 2000 °C, which means that it is highly energy demanding. Hydrometallurgical approaches are normally carried out at lower temperatures (between 90 °C and 200 °C), but they require the use of chemicals, such as HCl, H_2_SO_4_, HNO_3_, HClO_4_, and H_2_O_2_.^1,14^ The main problems of these techniques are connected to the toxic gas emissions (such as dioxins) and the acid wastewater generation, risking human health and environment.^16^ In the last decades, the interest in biotechnology has increased, as it is considered greener and more sustainable than the traditional chemical approaches, due to the lowest operative costs, energy demand, and waste production.^29–31^

In recent years, many researchers have studied the bioleaching approaches for metal recovery (mainly Cu, Zn, Al, Ni, and Co) from PCBs using iron-oxidizing bacteria (mainly *Acidithiobacillus ferrooxidans*) driven by the Fe^3+^ regeneration derived from the bacteria metabolism^28–40^ :^28–40^

1 $$\begin{array}{ll} {\mathrm{Cu}}^{0}+{2\mathrm{Fe}}^{3+}\to {\mathrm{Cu}}^{2+}+{2\mathrm{Fe}}^{2+} & \left(\mathrm{chemical reaction}\right) \end{array}$$

2 $$\begin{array}{ll} {\mathrm{Fe}}^{2+}+O_{2}+{4H}^{+}\to {\mathrm{Fe}}^{3+}+{2H}_{2}O & \left(\mathrm{bacteria metabolism}\right) \end{array}$$

Until now, the main limitations of this process underlined by the literature are slow kinetics and the relatively low quantity that can be bioleached due to toxicity problems. These aspects represent a big obstacle to make biotechnologies more attractive for stakeholders involved in process scale-up.^45^

In this context, the Copper BIOTECH (IT102023000002973) patent provides a solution based on bioleaching that overcomes the highlighted limits.^46^ This work presents a roadmap toward environmentally sustainable bioleaching technologies, based on the identification of bottlenecks (energy demand, length of the treatment, toxicity), of solutions (from stirred tank reactor to fixed bed column with adequate recirculation and makeover) and of tools (predictive mathematical models for simulation).

## 2 Materials and Methods

### 2.1 Preparation of the Printed Circuit Boards

PCBs used in this study are recovered from computers. After manual removal of the main parts of electronic components (the components of the upper and lower compound units, e.g., capacitors, batteries, and resistors), the material that composed the structure layer was shredded using stainless steel blades and pliers to obtain a granulometry less than 1.0 mm. Plastic and fiberglass were removed from pulverized PCBs by density separation, washing them twice with NaCl saturated water.^33,47^ The metal composition of the resulting pulverized PCBs is the following (w/w): Cu 20 ± 5%, Zn 2.0 ± 0.5%, Ni 0.6 ± 0.2%, and Al 1.2 ± 0.7%.

### 2.2 Assessment of the Carbon Footprint

The main aim of the environmental impact assessment of the process is the identification of the PCB concentration at which the bioleaching process results in the most sustainable extraction choice compared with primary production. The assessment referred to a bioreactor with a reaction volume of 1 L. The bioleaching approach considered for the analysis is the process reported by Becci et al., where PCB leaching by bacteria *At. ferrooxidans* is carried out in three phases: the first, where bacteria grow without PCBs; the second, when a known amount of the substrate is added to the medium; and the third, when 80% of the solution is replaced with fresh medium.^29^ The mass and energy balances used for the environmental evaluation impact (input and output) are reported by Becci et al. as concern biotechnological treatment (Scenario 1).^33^

The following step is the metal (Fe, Cu, and Zn) recovery process reported by Amato et al.^48^ In detail, Fe is recovered by precipitation with NaOH (∼35 g/L), Cu by cementation with Zn (Zn/Cu molar ratio = 1.1:1), and Zn by precipitation with oxalic acid (OA) (OA/Zn molar ratio = 1.3:1) (Scenario 1 ^48^). The positive effects of the recovery of secondary raw materials (Cu and Zn) are quantified as avoided impacts for primary production.^49^ The software LCA for Expert v. 10.9.0.31, integrated with My Professional Database (v.2024.2), is used for the production processes of energy and raw materials and quantification of the carbon footprint.

### 2.3 Column Design and Leaching Experiment

Cu extraction from PCBs is carried out in a continuous column setup. The column consisted of a 100 mL plastic cylinder. At the bottom, the column was connected to the inflow solution tank by an injection inlet pipe. At the top, the outflow solution went back to the beaker through an outlet pipe. The column dimensions were 20 cm height and 2.8 cm internal diameter. The upward flow is assured by a peristaltic pump (Figure 1a). The column was initially filled with 200 g of washed PCB powder with a granulometry lower than 1 mm. The packed column showed a ε, the porosity value, of around 0.55 (percentage of empty spaces between the solid matrix particles). The top of the column is covered with glass wool to prevent PCB leakage due to the flow of solution.

**Figure 1:**
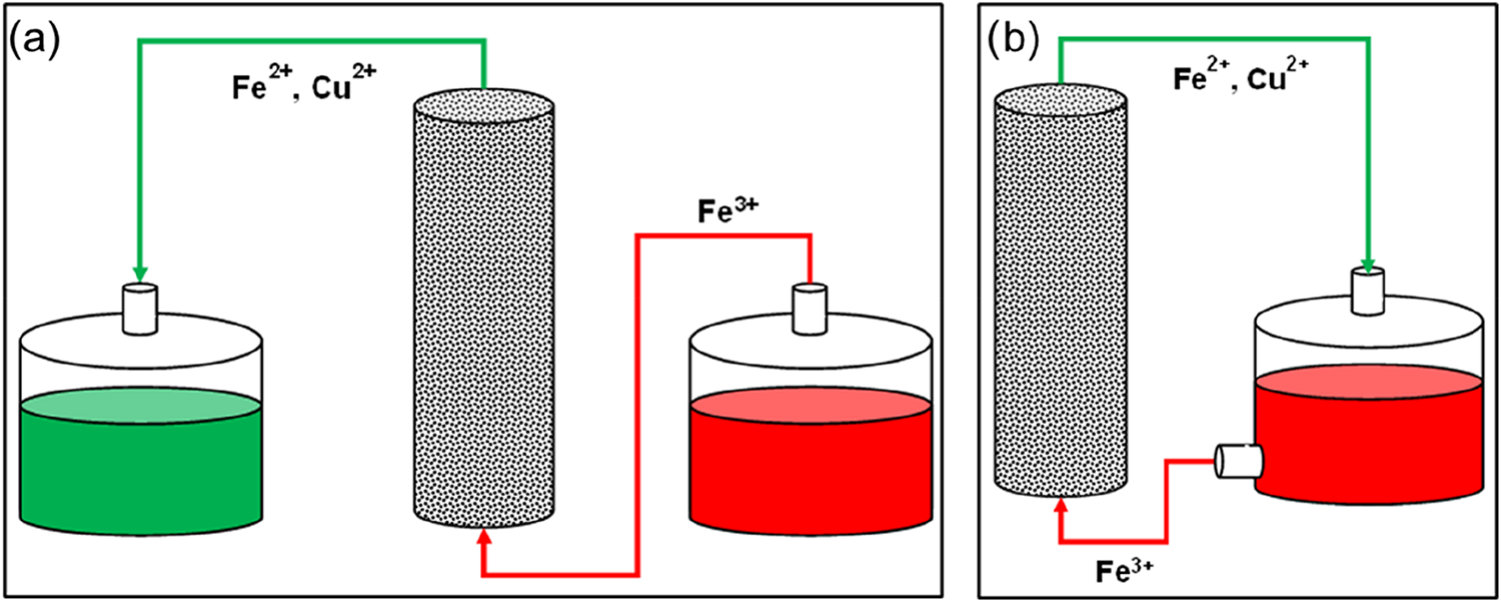
Schematic representation of the column setup for Cu chemical leaching with ferric iron (a) and for bioleaching (b). The schema of the bioleaching process carried out in the column setup is similar to that reported by other authors.^45,50^

The leaching solution is composed of Fe^3+^ solution with an iron concentration of around 10 g/L and a pH of 1.5. Two different peristaltic pump velocities are tested (25 and 5 mL/min), meaning a rate of 4.06 and 0.81 cm/min and a contact time of 5 and 25 s, respectively. The chemical leaching was carried out to verify leaching kinetics and to adapt the developed mathematical model at the new leaching design.^14^ The Cu^2+^, Fe^3+^, and Fe^2+^ concentrations in the column are described by the modified differential equations reported by Becci et al.^17^ (eqs 3–5):

3 $$\frac{d{\mathrm{Cu}}_{t\left(\mathrm{col}\right)}^{2+}}{dt}=\frac{Q\cdot {\mathrm{Cu}}_{t\left(\mathrm{in}\right)}^{2+}+k\cdot \left({\mathrm{Cu}}_{t}^{0}-{\mathrm{Cu}}_{t}^{2+}\right)\cdot {\mathrm{Fe}}_{t}^{3+}\cdot V-Q\cdot {\mathrm{Cu}}_{t\left(\mathrm{out}\right)}^{2+}}{V}$$

4 $$\frac{d{\mathrm{Fe}}_{t\left(\mathrm{col}\right)}^{3+}}{dt}=\frac{Q\cdot {\mathrm{Fe}}_{t\left(\mathrm{in}\right)}^{3+}-m\cdot k\cdot \left({\mathrm{Cu}}_{t}^{0}-{\mathrm{Cu}}_{t}^{2+}\right)\cdot {\mathrm{Fe}}_{t}^{3+}\cdot V-Q\cdot {\mathrm{Fe}}_{t\left(\mathrm{out}\right)}^{3+}}{V}$$

5 $$\frac{d{\mathrm{Fe}}_{t\left(\mathrm{col}\right)}^{2+}}{dt}=\frac{Q\cdot {\mathrm{Fe}}_{t\left(\mathrm{in}\right)}^{2+}+m\cdot k\cdot \left({\mathrm{Cu}}_{t}^{0}-{\mathrm{Cu}}_{t}^{2+}\right)\cdot {\mathrm{Fe}}_{t}^{3+}\cdot V-Q\cdot {\mathrm{Fe}}_{t\left(\mathrm{out}\right)}^{2+}}{V}$$

where *Q* (mL/min) is the flow rate of the peristaltic pump; Cu_*t*(in)_^2+^, Fe_*t*(in)_^3+^, and Fe_*t*(in)_^2+^ are Cu^2+^, Fe^3+^, and Fe^2+^ concentrations in the inflow solution (mol/L), respectively; *k* is the rate constant determined by Becci et al.;^44^ *m* is the Fe^3+^/Cu stoichiometric coefficient (2.0); *V* is the free volume of full column with PCBs (0.068 L); Cu_*t*_^0^ and Cu_*t*_^2+^ are the Cu concentration in PCB powder by the time and the Cu concentration in the leaching solution inside the column, respectively (mol/L); Fe_*t*_^3+^ is the Fe^3+^ concentration in the leaching solution inside the column (mol/L); Cu_*t*(out)_^2+^, Fe_*t*(out)_^3+^, and Fe_*t*(out)_^2+^ are the Cu^2+^, Fe^3+^, and Fe^2+^ concentrations in the outflow solution (mol/L).

### 2.4 Optimization of the Patented Technology Copper BIOTECH with the Mathematical Model

The patented technology Copper BIOTECH (IT102023000002973) provides a design of the bioleaching like chemical leaching carried out in the column setup.^46^ The main difference is that, after passing through the column, the leaching solution returns to the bioreactor, where bacterial strain *At. ferrooxidans* oxidizes Fe^2+^ back to Fe^3+^ (eq 2). This regenerated Fe^3+^ can participate again in the chemical reaction with Cu in the PCB powder (eq 1) (Figure 1b).

The optimization of the patented technology is driven by the mathematical model adapted for the new column design, integrating previous model with the mathematical model to describe the bacteria metabolism developed by Becci et al.^44^ To describe the bacteria abundance (*N_t_*) and the Fe^2+^, Fe^3+^, and Cu^2+^ concentrations in the bioreactor, the following differential equations are used (eqs 6–9):

6 $$\frac{{dN}_{t}}{dt}=\mu \cdot N_{t}-\mu_{d}\cdot {\left({Fe}_{t\left(Biorea\right)}^{3+}\right)}^{2}\cdot N_{t}-\mu_{tox}\cdot {\left({Cu}_{t\left(Biorea\right)}^{2+}\right)}^{2}\cdot N_{t}$$

7 $$\frac{{dFe}_{t\left(Biorea\right)}^{2+}}{dt}=\frac{-\frac{\mu }{Y_{NS}}\cdot N_{t}\cdot V_{rim}+Q\cdot {Fe}_{t\left(out\right)}^{2+}\cdot {\mathrm{AW}}_{Fe}}{V_{Biorea}}$$

8 $$\frac{{d\mathrm{Fe}}_{t\left(\mathrm{Biorea}\right)}^{3+}}{dt}=\frac{\frac{\mu }{Y_{\mathrm{NS}}}\cdot N_{t}\cdot V_{\mathrm{rim}}+Q\cdot {\mathrm{Fe}}_{t\left(\mathrm{out}\right)}^{3+}\cdot {\mathrm{AW}}_{\mathrm{Fe}}}{V_{\mathrm{Biorea}}}$$

9 $$\frac{{\mathrm{Cu}}_{t\left(\mathrm{Biorea}\right)}^{2+}}{dt}=\frac{{\mathrm{Cu}}_{t-1}^{2+}\cdot V_{\mathrm{rim}}+Q\cdot {\mathrm{Cu}}_{t\left(\mathrm{out}\right)}^{2+}\cdot {\mathrm{AW}}_{\mathrm{Cu}}\cdot \Delta t}{V_{\mathrm{Biorea}}}$$

where μ_d_ and μ_tox_ are the toxicity parameters of Fe^3+^ and Cu^2+^ on bacteria growth, respectively, *Y*_NS_ is the bacteria yield on the substrate, these three parameters are determined in the previous work by Becci et al.;^44^ Fe_*t*(Biorea)_^2+^, Fe_*t*(Biorea)_^3+^, and Cu_*t*(Biorea)_^2+^ are the Fe^2+^, Fe^3+^, and Cu^2+^ concentrations in the bioreactor (g/L); Fe_*t*(out)_^2+^, Fe_*t*(out)_^3+^, and Cu_*t*(out)_^2+^ are the concentrations in the outflow leaching solution (g/L); AW_Fe_ and AW_Cu_ are Fe and Cu atomic weights; *V*_Biorea_ is the volume of the bioreactor (3.6 L), Δ*t* is the selected interval time (0.1 min), while *V*_rim_ is the volume of the bioreactor given by the following equation (eq 10):

10 $$V_{\mathrm{rim}}=V_{\mathrm{Biorea}}-Q\cdot \Delta t$$

*V*_rim_ was used to calculate and estimate the concentrations of metals (Cu^2+^, Fe^2+^, and Fe^3+^) within the bioreactor, accounting for the dilution of these metal concentrations inside the system due to the outflow solution from the column.

On the other hand, μ is the specific growth rate, and it is given by the Monod model (eq 11):

11 $$\mu =\frac{\mu_{\max}\cdot {\mathrm{Fe}}_{t}^{2+}}{K_{S}+{\mathrm{Fe}}_{t}^{2+}}$$

where μ_max_ (1/min) is the maximum specific growth rate and *K*_S_ (g/L) is the substrate saturation constant.

### 2.5 Analytical Determination

Leaching solutions are periodically analyzed for the determination of the Cu concentration, carried out by an atomic absorption spectrophotometer (AAS) (Varian spectrometer SpectrAA 200). On the other hand, Fe^3+^ and Fe^2+^ detections are performed by an UV/VIS spectrophotometer by the colorimetric thiocyanate method (Jasco Model 7850).^33,51–54^

## 3 Results and Discussion

### 3.1 Environmental Impact Assessment

The first target of this study is to identify the best operative conditions for bioleaching in a stirred tank reactor. The environmental sustainability analysis of the process was conducted based on optimal conditions identified in previous studies, both for the bioleaching phase—characterized by Cu and Zn leaching efficiencies exceeding 95%—and for the selective metal recovery phase, where both recovery and purity efficiencies for Cu and Zn were above 95%.^33,48^ A Monte Carlo simulation (around 8000 simulations), considering an uncertainty of the characterization factors from the Gabi data set (kg of CO_2_-equiv) of 10%, is carried out. Data used in the environmental analysis were retrieved from previous studies and reweighed for the new functional unit (1 L of bioreactor; Table 1). In order to identify conditions under which the biotechnological process results in a lower environmental impact compared with primary metal production, specifically when environmental credits from avoided primary production of Cu and Zn exceed the impacts associated with raw material use and energy demand for metal recovery from PCBs, thus yielding a negative net impact, a Monte Carlo analysis was conducted by varying three key parameters: concentration of PCBs in the biotechnological process and concentrations of Cu and Zn within PCBs (Table 1).

**Table 1:** Input Data for the Environmental Sustainability Analysis (Functional Unit 1 L of Bioreactor) in the Monte Carlo Simulation^33,48^

scenario step	fixed input data	variable input data	range
leaching	electricity 0.132 kWh		
	FeSO_4_ 2.5 g		
	water 0.17 kg		
	H_2_SO_4_ 0.015 kg		
	NaOH 0.01 kg		
	micronutrients 0.4 g		
		PCB concentration	50–200 g/L
recovery	NaOH 0.035 kg		
	Zn/Cu molar ratio 1.1:1		
		Zn concentration	4–45 g/L
	OA/Zn molar ratio 1.3:1		
		OA concentration	7–78 g/L
	energy 4.7 × 10^–4^ kWh		
credit		Cu recovered	3.5–40 g
		Zn recovered	0.5–6.5 g

The results show that, at the selected conditions, bioleaching process has a higher environmental load than the primary production (Figure 2), considering all of the possible Cu and Zn concentrations reported in literature (green points).^32–44^ Furthermore, the results show that the bioleaching environmental impact in the global warming category decreases with PCB concentration growth in the bioleaching process and using a PCB composed of high Cu concentration (Table 2). As reported in Figure S1, the main critical step is the metal recovery (45% of the whole environmental load), mainly due to Fe and Cu recoveries. Recovery is the phase with the highest environmental impact, despite the PCB or Cu starting concentrations (Table 2). Indeed, increasing the concentration of PCBs and/or Cu leads to a greater demand for materials required in the Cu recovery step, which in turn increases the materials needed for Zn recovery, ultimately resulting in a higher environmental burden associated with the selective metal recovery phase.

**Figure 2:**
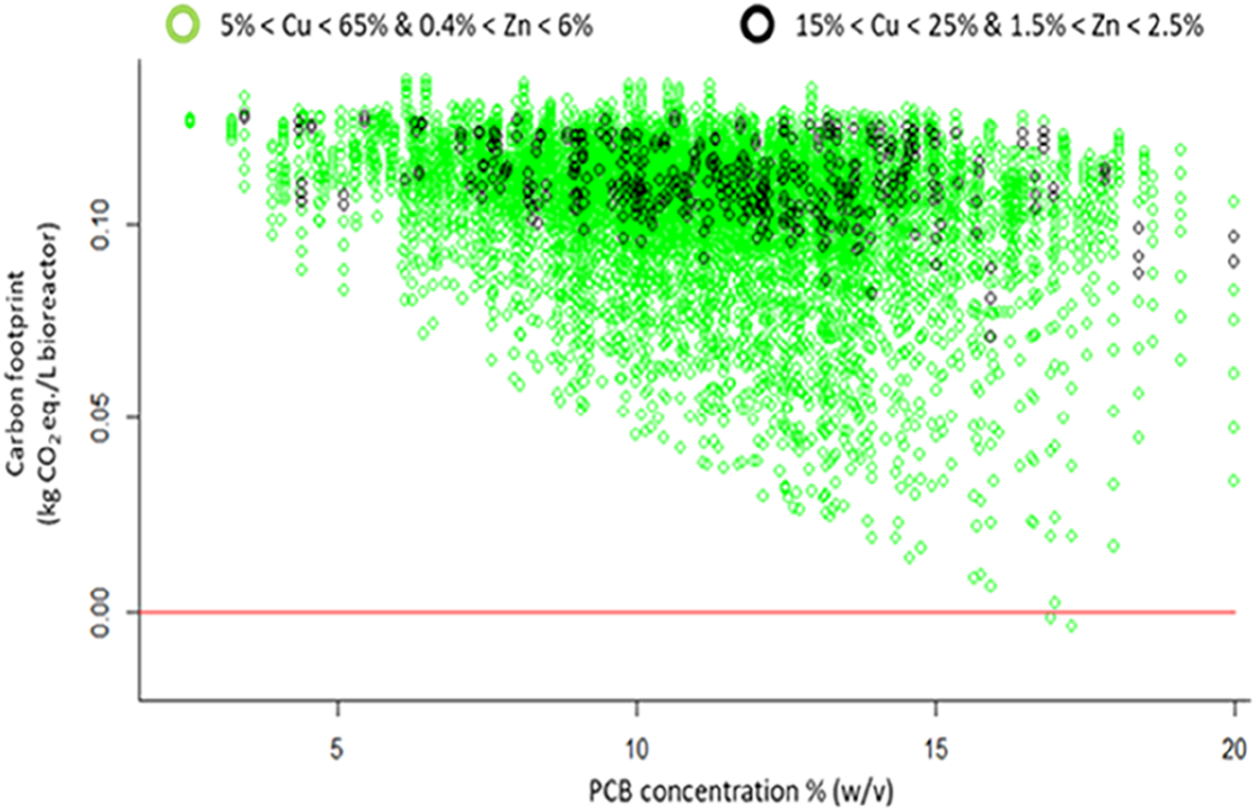
Carbon footprint of the metal extraction process from electronic waste as a function of PCB and Cu/Zn concentrations (output of the Monte Carlo simulation).

**Table 2:** Percentage Repartition of the Environmental Load Considering the Different PCB and Cu Concentrations in the Monte Carlo Analysis

		PCB concentration (w/v)					
		5 ± 2		10 ± 2		15 ± 2	
Cu concentration	step	mean	SD	mean	SD	mean	SD
10 ± 5	leaching	38	3	32	3	27	3
	recovery	45	1	45	1	45	1
	credit	17	3	23	3	28	3
20 ± 5	leaching	32	3	25	3	20	2
	recovery	45	1	45	1	45	1
	credit	23	3	30	3	35	2
50 ± 5	leaching	20	2	14	1	11	1
	recovery	45	1	45	1	45	1
	credit	35	5	41	2	44	1

On the other hand, the results demonstrate that in the bioleaching step, the main criticality is the energy demand due to the long time required (Figure S1). Therefore, results suggest that a relevant environmental impact decrease could be achieved combining a decrease of energy demand in the bioleaching process, decreasing the time required to complete the metal extraction, and a change in the metal recovery process. Indeed, the credit connected to Zn represents only 5% of the environmental gain, which is not enough to balance the impact of its recovery (Figure S1). And so, a possibility is the Cu recovery with electrodeposition, thereby eliminating the Zn recovery step. Nevertheless, the present paper focuses only on the carbon footprint, and further assessments could take into account additional impact categories, able to quantify the effects connected to wastewater production and gas emission by primary production processes.

### 3.2 Chemical Leaching with the Column Setup

Considering the results of the Monte Carlo simulation, an alternative leaching technology in a column setup was considered to reduce the time required to complete the bioleaching process. A first set of experiments and simulations were carried out for the chemical leaching with Fe^3+^, to find the best conditions and to verify if the modified mathematical model (eqs 3–5) could successfully predict the experimental results. Two different flow rates were tested (25 and 5 mL/min). Using the highest flow rate (25 mL/min) (Figure 3a), we can see that the contact time between solution and powder PCBs in the column is not high enough to complete the chemical reaction (eq 1). More in detail, we can see that the outflow Fe^3+^ concentration starts to increase from 0.5 g/L in the first 5 min to 2.0 g/L after 2 h: this is a demonstration that not all Fe^3+^ is reacting with Cu inside PCBs. On the other hand, using the lowest flow rate (5 mL/min), Fe^3+^ in the outflow is always lower than 1.0 g/L in the first 31 h (Figure 3b). This demonstrates that there is enough time to complete the reaction between Fe^3+^ and Cu inside the column (eq 1). In this case, we have a constant Cu concentration in the outflow around 4 g/L, from 8 to 33 h.

**Figure 3:**
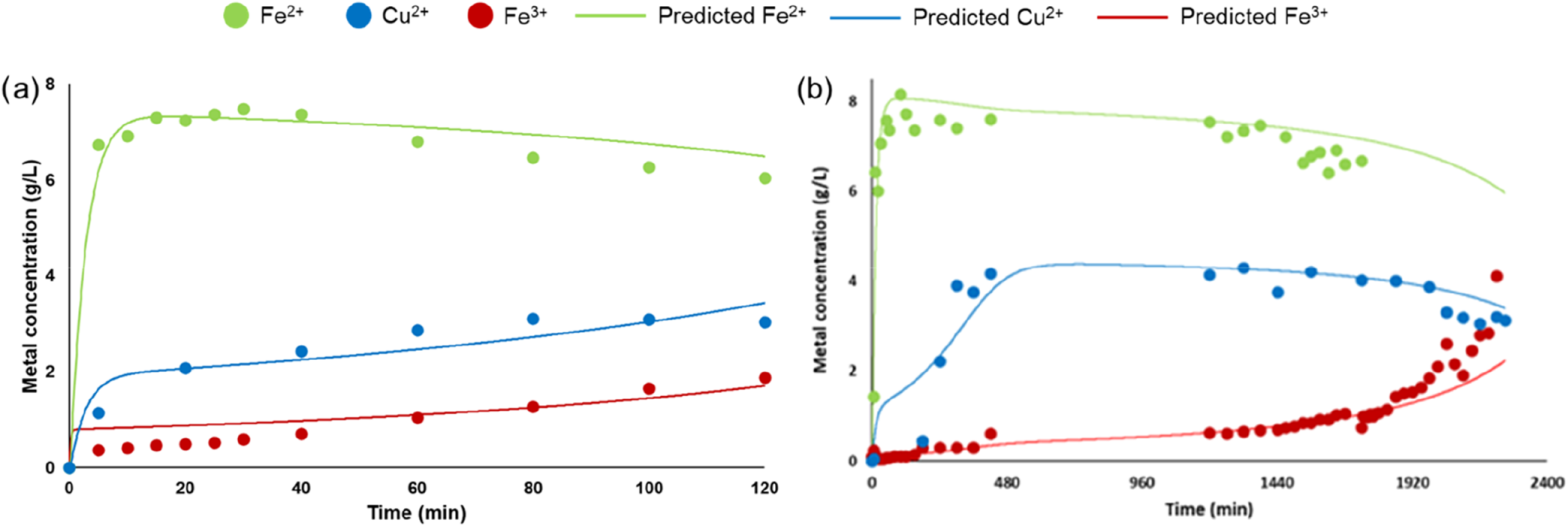
Metal (Fe^3+^, Fe^2+^, and Cu) concentrations in the outflow solution in the leaching test with the column design considering the two flow rates: 25 mL/min (a) and 5 mL/min (b). Lines were predicted through eqs 3–5.

After 38 h, the end of the experiment, around 43 g of Cu was extracted with a leaching efficiency of 85%, with a Cu extraction rate of 1.15 g/h (Figure S2). Data in Figure 3 also evidence the excellent agreement among the predictive model (eqs 3–5) and experimental data, providing a powerful tool for optimization purposes. Finally, we also verified the performance of the model when the outlet solution was recirculated after a first column leaching.

The test with the highest flow rate (25 mL/min) was replicated, and the outlet solution collected after 2 h was refed to the column (Figure 4). Indeed, as previously observed, this solution still has Fe^3+^ potentially useful to react with the residual Cu in the column. This test is fundamental, being the recirculation of solution an important aspect for Copper BIOTECH patented technology. As a whole, the achieved results confirm that the mathematical model successfully predicts Cu leaching, being in excellent agreement with the experimental data (*R* ^2^ higher than 0.9 for all tested conditions, without any fitting procedure applied).

**Figure 4:**
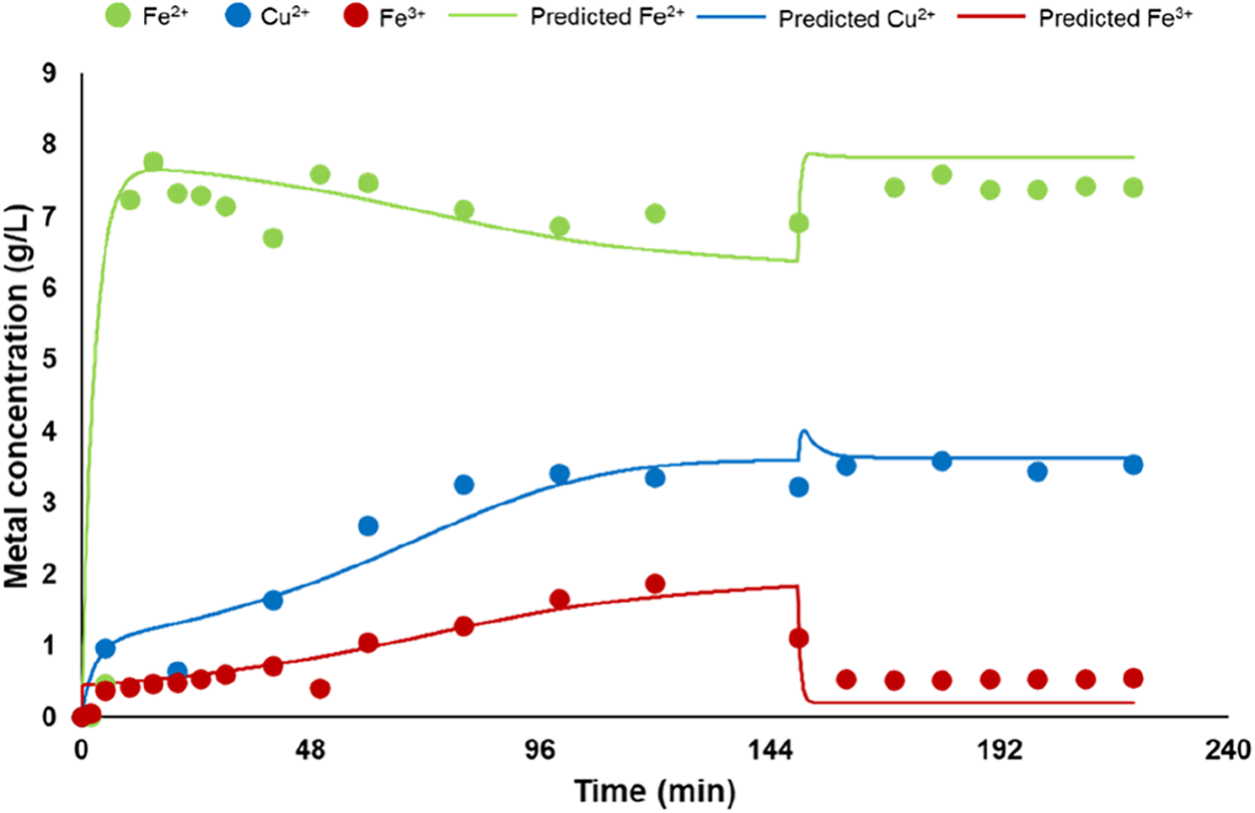
Metal (Fe^3+^, Fe^2+^, and Cu) concentrations in the outflow solution in the leaching test with the column design considering the solution recirculation, flow rate 25 mL/min. Lines were predicted through eqs 3–5.

### 3.3 Optimization of the Bioleaching Process Using the Mathematical Model in the New Column Design

Starting from these results, the mathematical model (eqs 6–11) was used to find the best operative conditions in the bioleaching process. We start from the conditions reported by Becci et al.,^29,44^ with a first phase dedicated to bacterial (*At. ferrooxidans*) growth without PCBs; after 48 h, the solution is fed to the column with a flow rate of 5 mL/min. After the passage through the column, the solution flows back to the bioreactor, where the Fe^2+^ produced by the chemical reaction with Cu is oxidized again by bacteria metabolism to Fe^3+^ (Figure 1b). After 48 h, 80% of the leaching solution is removed and replaced by new fresh medium (Fe^2+^), to reduce the Cu concentration and so the metal toxicity on bacteria metabolism.

The simulated results from the mathematical model (eqs 6–11) show that Fe^2+^ and Cu^2+^ in the bioreactor start to increase when the solution passes through the column after bacterial growth for 48 h (Figure 5a). The Cu^2+^ increase is fairly constant in the first 48 h, up to a stationary value around 8 g/L. The Cu extraction recorded a rate of 0.66 g/h in the first 24 h, and this extraction rate decreases to 0.47 g/h in the second 24 h. The Cu leaching efficiency at the end of the first phase was around 60% (Figure S3a).

**Figure 5:**
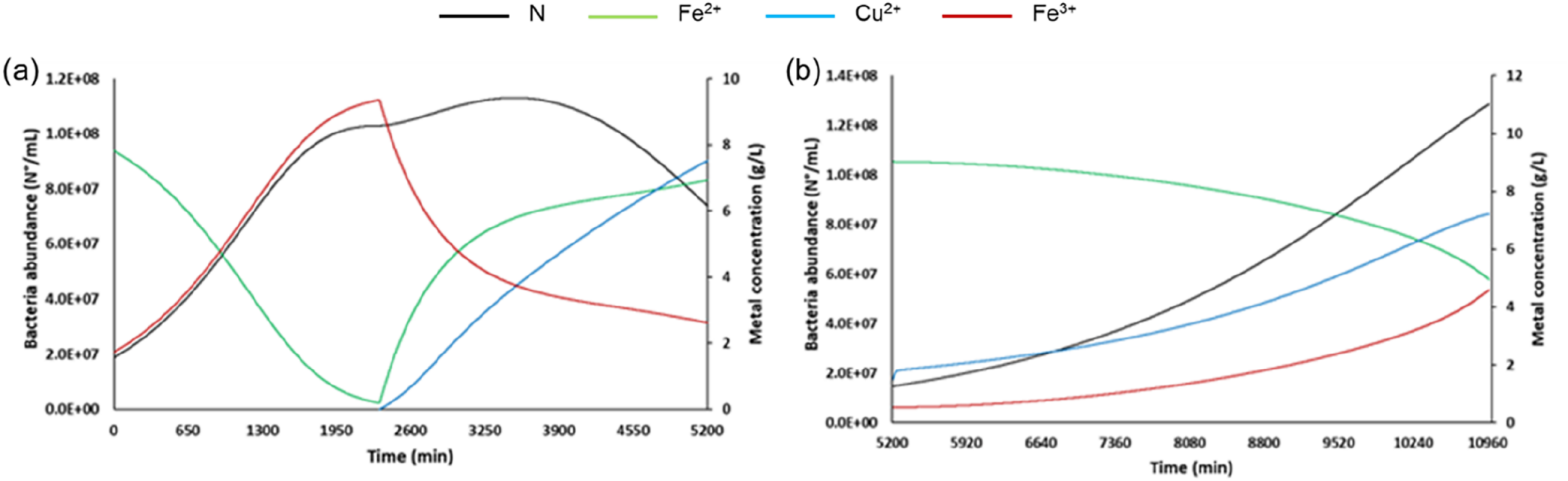
Temporal profile of Fe^2+^, Fe^3+^, Cu^2+^, and bacteria number in the bioreactor in the first (a) and second phases (b) for the first simulation by eqs 6–11 (see text for details).

The main critical issues identified in this first leaching phase are decreases in Fe^3+^ and bacterial abundance inside the bioreactor. Copper BIOTECH patent is based on constant bacterial activity in order to balance the Fe^2+^ input from the column and the Fe^2+^ oxidized by bacteria metabolism. In the first phase, this objective is not achieved. More in detail, the Fe^2+^ input from the column is around 10 times higher than the Fe^2+^ oxidized by bacteria metabolism (Figure S4a). For this reason, the Fe^3+^ concentration in the bioreactor decreases from 9.4 to 3.5 g/L in the first 24 h and to 2.6 g/L at the end of the first leaching phase (after 48 h) (Figure 5a). Moreover, the fast Cu concentration increase in the bioreactor caused higher bacteria mortality due to the Cu toxicity and, therefore, a decrease of bacteria metabolism. As reported previously, concentrations between 4 and 9 g/L of Cu determined a 90% inhibition on bacteria activity.^45,55^

In the second phase, when 80% of the bioleaching solution from the bioreactor is removed, Fe^3+^ and Cu concentrations in the bioreactor decrease from 2.6 and 8 to 0.5 and 1.5 g/L, respectively. After 96 h (4 days) of the second phase, Fe^3+^ and Cu concentrations increase to 4.6 and 7.2 g/L, respectively (Figure 5b). At the end of the second leaching phase (96 h), a Cu leaching efficiency higher than 95% is achieved (Figure S3b). At the end of this phase, only around 50% of the whole Fe is in the oxidized form (Fe^3+^) (Figure 5b). In the first 24 h of this phase, the Fe^2+^ input from the column is 10 times higher than the Fe^2+^ oxidized by the bacteria metabolism (Figure S4b). This is mainly because at the beginning of this phase, the number of bacteria is relatively low, and so the bacteria metabolism is not active enough to balance the Fe^2+^ input from the column. For this reason, in the first 24 h, the Cu leaching rate is only 0.11 g/h and the amount of Fe^3+^ available for the chemical reaction with Cu is quite low in the column. After 24 h, the bacterial abundance starts to grow exponentially, and consequently, the bacterial metabolism starts to increase Fe^3+^ concentration and Cu leaching rate. Therefore, the ratio between the Fe^2+^ input and the Fe^2+^ oxidized by bacteria metabolism decreases from 10, after 24 h, to 2, at the end of 96 h (Figure S4b). Moreover, the Cu extraction rate increases from 0.11 g/h in the first 24 h to 0.15, 0.26, and 0.32 g/h in the second, third, and fourth 24 h, respectively. The model confirmed that the new design of the bioleaching technology allows to decrease the time from 11 days reported by Becci et al. to 8 days predicted by the model, with a consequent saving in energy demand.^29,44^

Considering the results of the first simulation and the main identified critical issues, a second simulation was carried out. The first two changes are

- the time when to start the solution recirculation, not at the end of the bacteria growth when the whole Fe is in the Fe^3+^ ions form, but when the bacteria metabolism reaches the maximum value (around 0.005 g/(L·min) of Fe^2+^ oxidized by bacteria metabolism), and so after 20 h, when the Fe^2+^ concentration is around 35% of the whole Fe;
- the flow speed is halved from 5 to 2.5 mL/min.

Thanks to these changes, in this first leaching phase, the bacterial abundance continues to grow and the bacteria metabolism rate is high enough to balance the needs for Fe^2+^ oxidation (Figure S5a). In this phase, a Cu extraction rate of 0.25 g/h is achieved. After 15 h, when the Cu concentration in the bioreactor is around 1 g/L, the flow rate of the solution is halved again (around 1.25 mL/min) to limit the Cu toxicity on bacteria metabolism and to increase the bacteria adaptation at the new environment. At the end of the first leaching phase, a final Cu concentration of 3.7 g/L is achieved (Figure 6a), with a Cu leaching rate of 0.22 g/h in the last 33 h and a final leaching efficiency of around 30% (Figure S6a). Under these conditions, the bacterial abundance remains constant during this phase, and the Fe^3+^ concentration continuously increases, up to a value of 7.5 g/L, around 80% of the whole Fe (Figure 6a).

**Figure 6:**
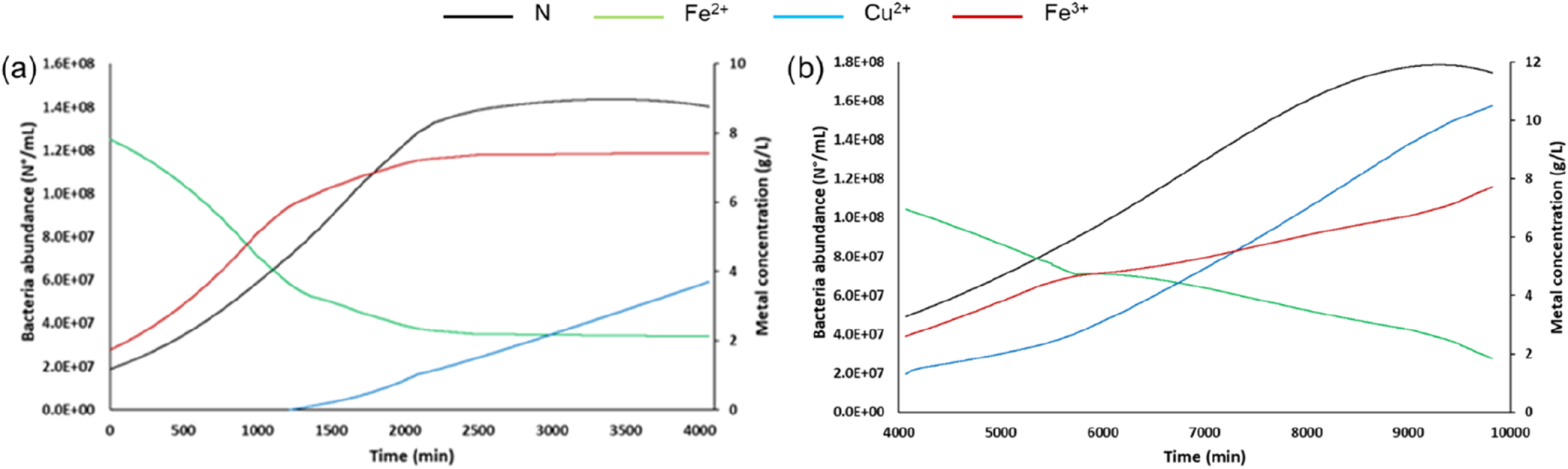
Temporal profile of Fe^2+^, Fe^3+^, Cu^2+^, and bacteria number in the bioreactor in the first (a) and second phases (b) for the second simulation.

In the second phase, it was proposed to remove 65% of the leaching solution instead of 80%. With this choice, it was expected to preserve bacterial activity. Therefore, with this alternative option, Fe^3+^ concentration, after the substitution of the leaching solution with fresh new medium, was around 2.5 g/L, and, consequently, Cu leaching rate at the beginning of the second phase was faster than in the first simulation (0.16 and 0.11 g/h, respectively). Moreover, for the first 27 h of the second phase, a feed flow rate of 1.25 mL/min was applied to the column, to allow for the increase in the bacterial abundance. When Fe^2+^ and Fe^3+^ concentrations are the same, the flow speed is doubled (2.5 mL/min). This new design allows one to obtain a Cu extraction rate of 0.44 g/h for the next 72 h (Figure 6b). Under these conditions, Fe^3+^ and Cu concentrations continue to increase up to 8 and 10 g/L at the end of this phase (96 h, 4 days), respectively, with a final Cu leaching efficiency higher than 95% (Figure S6b). As a whole, the achieved results allowed further reduction of the time of the treatment to 6.5 days.

The process redesign significantly reduced the specific energy demand, decreasing from 500 W/m^3^, typically required by mechanically stirred reactors, to 5 W through the implementation of a peristaltic pump system. This transition, coupled with a reduction in process time from 11 to 6.5 days as outlined in the Copper BIOTECH patent, resulted in an overall energy consumption decrease of approximately 70%. Notably, the enhanced energy efficiency achieved during the bioleaching phase contributed to an estimated 55% reduction in the total carbon footprint of the process (Figure 7).

**Figure 7:**
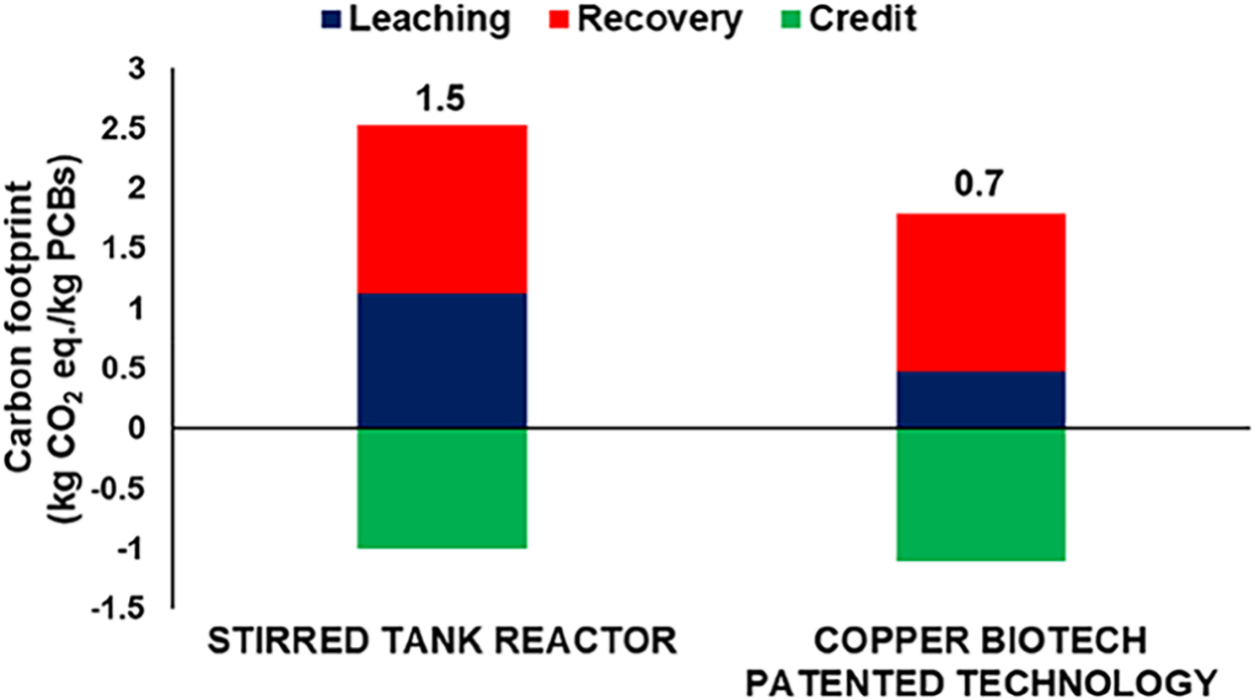
Carbon footprint of the copper recycling from PCB through bioleaching: stirred tank reactor vs the Copper BIOTECH patented technology (note: copper recovery is achieved through cementation with zinc, based on Amato et al., 2020; the credit associated with gold and silver is not included in the assessment).

## 4 Conclusions

The initial outcome of this study, focused on assessing the environmental sustainability of the bioleaching process for Cu recovery from PCBs, revealed that, even when the PCB loading was increased, the stirred bioreactor configuration did not offer advantages over primary Cu production. The analysis identified the main critical points as the selective metal recovery step and the high energy demand associated with the leaching process. To address these limitations, the Copper BIOTECH patent proposes an alternative reactor design aimed at reducing both the leaching time and the energy consumption by implementing a column bioleaching configuration. The present work showed that the integration of sustainability assessment (through LCA), mathematical models, and experiments could be useful to optimize an innovative process. The combination of these three approaches allowed us to decrease more than 50% the carbon footprint of the bioleaching process for the Cu recovery from PCBs, making it highly promising for the European economy, which currently promotes effective urban mining strategies.

## Supplementary Material



## Notes

Impact of bioleaching process is distributed among the different steps (Figure S1), the Cu leaching efficiencies (both experimental and simulated), for chemical (Figure S2) and bioleaching (Figures S3–S6) experiments, and the trend of ratio between Fe^2+^ input from the column and Fe^2+^ oxidized by the bacteria metabolism (Figures S4 and S5) (PDF)
