# Supplementary material for: Sustainable Optimization of Biotechnology for Cu Recovery from Printed Circuit Boards
Source: ACS Omega. 2025 Sep 3;10(36):41190–9. doi: 10.1021/acsomega.5c03870 (PMC12444616; doi:10.1021/acsomega.5c03870)
Supplement: Supplementary file 1 [file ao5c03870_si_001.pdf]

Supporting Information

Sustainable optimization of a biotechnology for Cu recovery from printed circuit boards

Alessandro Becci[a]*, José Miguel Rodríguez-Maroto[b], Juan Manuel Paz-Garcia[b], Francesca Beolchini[a], Alessia Amato[a]

[a]*Department of Life and Environmental Sciences, Università Politecnica delle Marche, Via Brecce Bianche, Ancona, 60131, Italy*
[b]*Department of Chemical Engineering, University of Malaga, Malaga, 29071, Spain*
**Corresponding author: a.becci@univpm.it*

Abstract

The increase of WEEE, rich in valuable elements, has pushed research towards the development of sustainability treatments for its exploitation. The high Cu concentration within PCBs (around 20-25% w/w) makes them a promising secondary resource. The aim of this work is the optimization of the patented bioleaching process driven by the minimization of the environmental load in the global warming category. The first environmental assessment was carried out considering bioleaching in a stirred tank reactor, also including metal recovery operations, using the best conditions identified in previous works. A mathematical model for bioleaching prediction was integrated inside the LCA methodology and used in Montecarlo simulations: this assessment highlighted the energy demand as main criticality. Consequently, a fixed bed column leaching was investigated both experimentally and theoretically through a predictive model, starting with the chemical leaching with Fe^{3+} and recirculation, as preparatory to our Copper BIOTECH patented technology. The excellent agreement among the predictive model and the experimental data provided a powerful tool for optimization purposes. The mathematical model was applied to patented technology and simulations allowed to identify the best operative conditions in bioleaching. The innovative design allows to decrease around 70% of energy demand and around 55% of the impact in the global warming category. Our study is fundamental to boost the application of sustainable bioleaching technologies in the world. Moreover, our methodological approach represents a guideline to meet sustainability goals within circular economy approaches for strategic metals.

Keywords: PCB, bioleaching, mathematical model, process optimization, carbon footprint

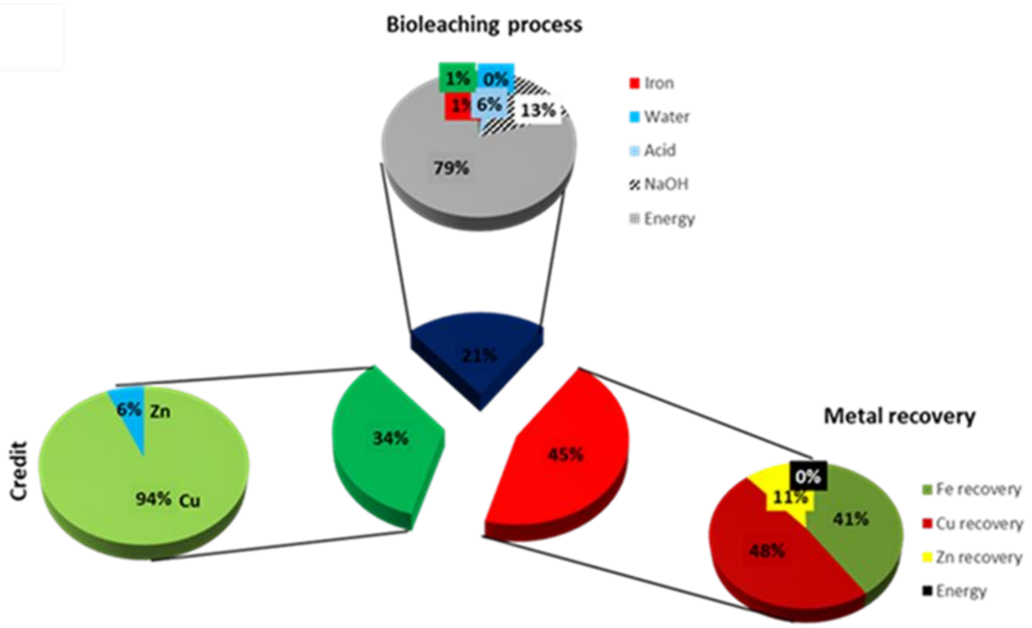

Figure S1: Distribution of the environment load among the different steps for the metal extraction.

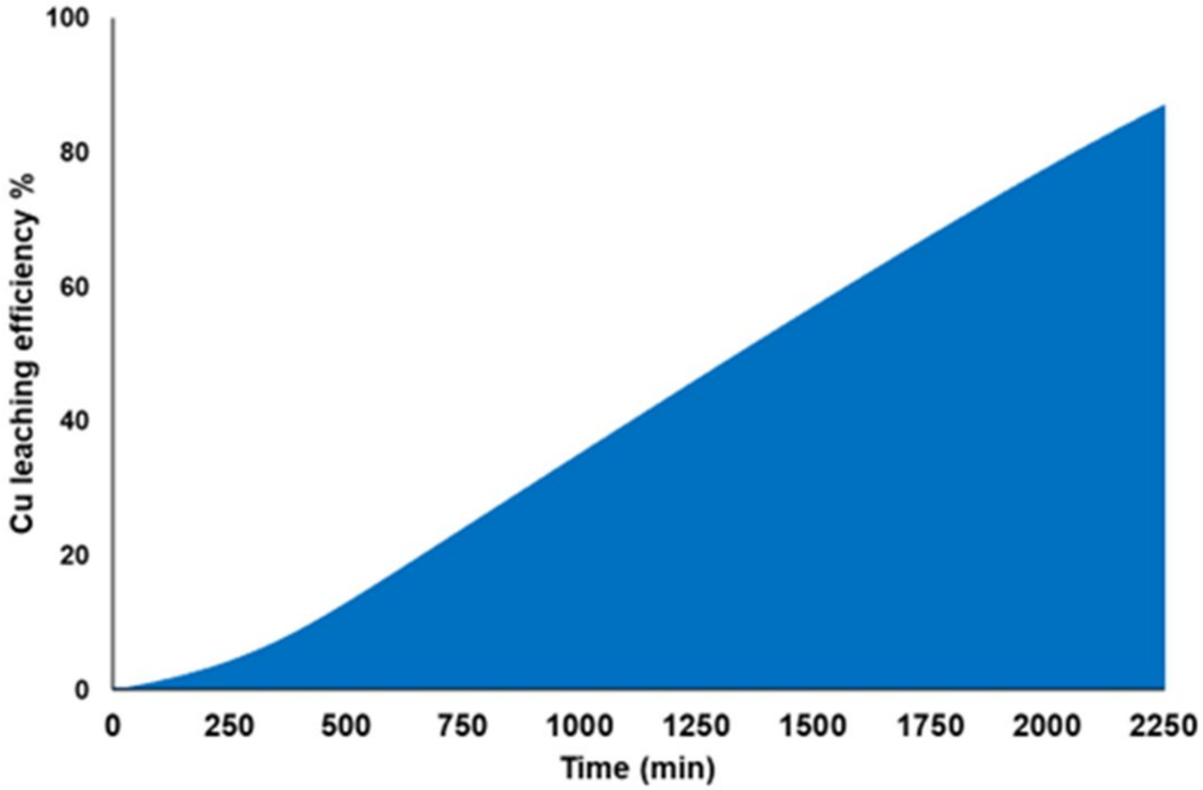

Figure S2: Cu leaching efficiency in the column set up with 5 mL/min flow rate, as predicted by equations (3) to (5).

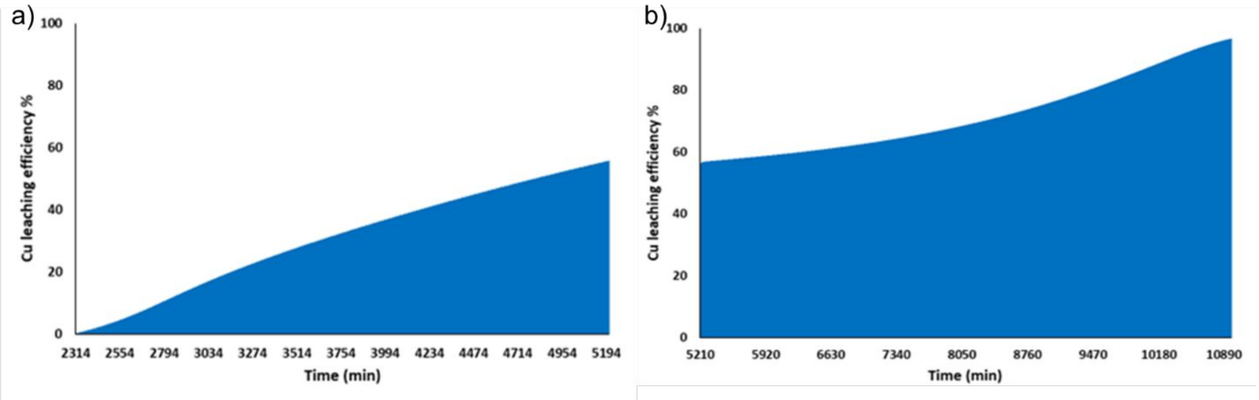

Figure S3: Cu leaching efficiency in the first (a) and in the second (b) phase for the first simulation.

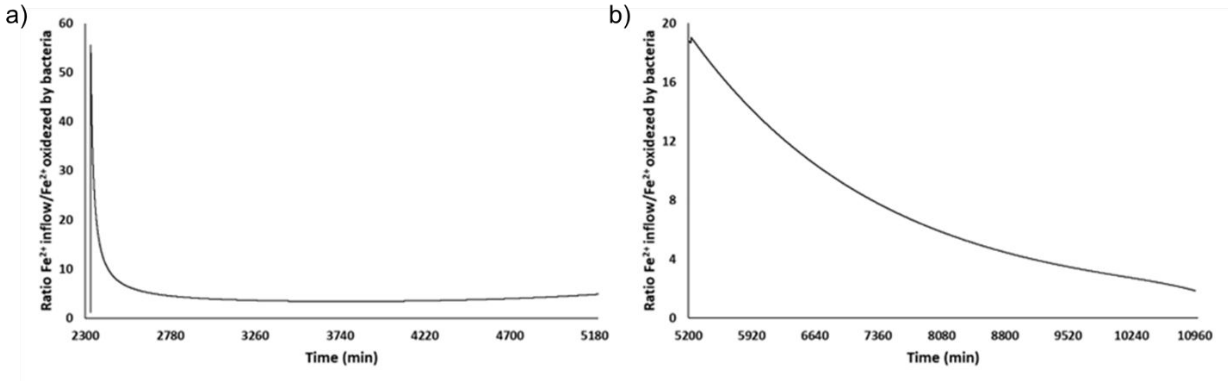

Figure S4: Ratio between input Fe^{2+} from the column and Fe^{2+} oxidized by bacteria metabolism in the first (a) and in the second (b) phase of the first simulation.

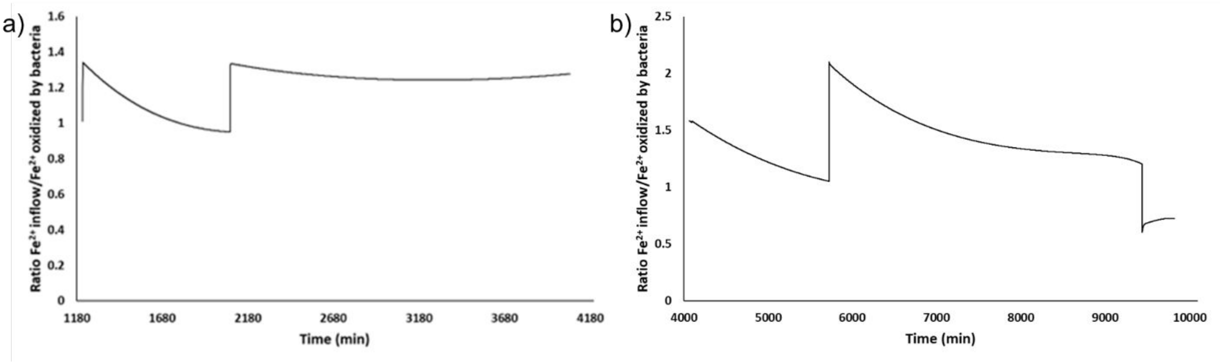

Figure S5: Ratio between input Fe^{2+} from the column and Fe^{2+} oxidized by bacteria metabolism in the first (a) and in the second (b) phase of the second simulation.

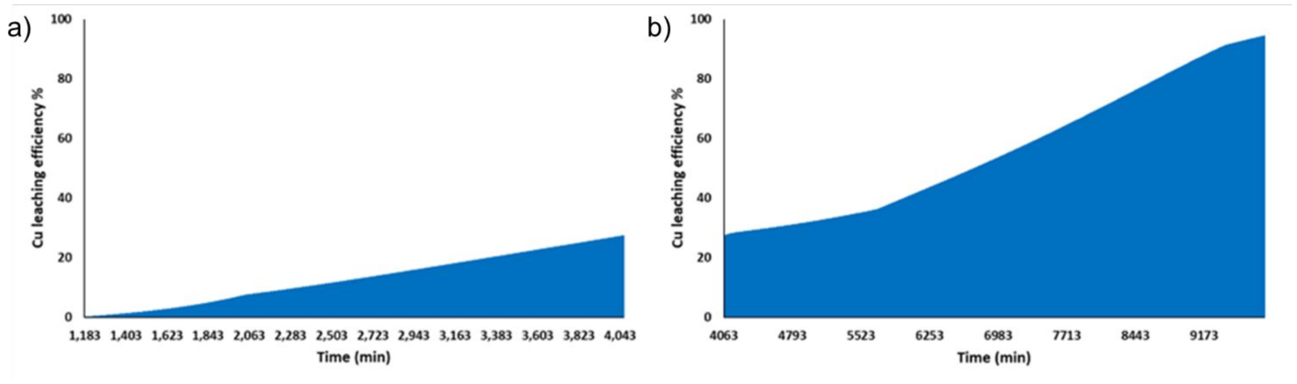

Figure S6: Cu leaching efficiency in the first (a) and in the second (b) phase for the second simulation.
